# StudentKost: a cross-sectional study assessing college students’ diets: reason for concern?

**DOI:** 10.1017/jns.2020.33

**Published:** 2020-09-03

**Authors:** Erlend L. Valen, Dagrun Engeset, Nina C. Øverby, Elisabet R. Hillesund

**Affiliations:** Department of Nutrition and Public Health, Faculty of Health and Sport Sciences, University of Agder, PO Box 422, 4604 Kristiansand, Norway

**Keywords:** Norway, Food consumption survey, Dietary intake, Young adults, Preconception health

## Abstract

College students constitute a significant proportion of the young adult population in Norway. They are in their reproductive years, which is of interest regarding diet and preconception health. Our objective was to assess young college students’ diet and nutrient intake in relation to national dietary recommendations and assess the probability of inadequate micronutrient intake for both genders using the Nordic Nutrition Recommendations, and also to evaluate its consequences on preconception health and create a groundwork for future interventions on this group. At the University of Agder (UiA), we enrolled 622 students aged 18–40 years for a cross-sectional study of student's diet, StudentKost. The students completed a food frequency questionnaire, including questions of supplement use, over the past 4 weeks. Intake of fruits, vegetables, oily fish, and whole grain was lower than recommended, as were mean intake of folate, iron, and iodine. Our main findings are that students have a somewhat suboptimal diet compared to the Norwegian dietary guidelines. Male students had generally lower diet quality than females. Compared to the Nordic Nutrition Recommendations (NNR), we also saw a relatively high probability of inadequate intake of several micronutrients and a very high probability for some micronutrients in a significant portion of the sample. Public health effort should be directed towards improving students and young adults’ diet in general, and interventions towards improving preconception health should be explored. The low participation rate limits the generalizability of our findings. Our findings encourage further investigation into young adults’ diet.

## Introduction

Diet strongly influences human health. Over the years, there has been a shift from exploring deficient intake of single nutrients to explore the importance of overall diet for human development and health^(1)^. Based on this research, evidence-based national food-based dietary guidelines have been developed, as well as age- and gender-specific recommendations for nutrient intake. The Norwegian health authorities survey population diet at regular intervals. Norkost 3, a nation-wide dietary survey carried out in 2011, indicated that the adult Norwegian population mainly had nutrient intakes within recommendations, but that a relatively large proportion did not comply with the dietary guidelines for many of the core food groups^(2)^. In general, there has been limited research on young adults’ diet in Norway, and Norkost 3 from 2011 is the only nation-wide food consumption survey that has been completed on adults since the Norkost 2 survey in 1997^(3)^.

More knowledge regarding young adults’ diet and dietary behaviour is warranted because new research has demonstrated associations between lifestyle and diet before conception and subsequent children's development and health^(4)^. The focus has mainly been on the mothers’ diet during pregnancy as the first step in affecting child health. Recently, the focus is expanded to include adolescents and young adults’ diet in the years and months before parenthood^(4–6)^. Which again is leading to increased awareness around a topic that has barely been addressed, i.e. how fathers’ diet and behaviour before conception can affect their children's health. Paternal obesity and undernutrition are both associated with increased DNA damage and endocrine misregulations^(5)^, while sperm motility could be positively affected by a healthy diet^(7)^. According to Frey *et al*., improving men's preconception health is important in conjunction with a healthy pregnancy, both directly and indirectly, including improving pregnancy outcomes and health practices as well as improving capacity for parenthood^(8)^.

Although a healthy diet is especially important during pregnancy to ensure a sufficient intake of vital micronutrients^(9)^, an optimal micronutrient status should ideally be acquired in advance^(4)^. Stephenson *et al*. are concerned that many women in low-, medium- and high-income countries will not be nutritionally prepared for pregnancy^(4)^. Public health interventions targeting dietary behaviour ahead of pregnancy may be a path to proceed. Barker *et al*. argue that although it is too early to draw definite conclusions about the long-term effect of pre-pregnancy diet interventions, the potential health benefits will be so high, and the costs so low, that research on measures on how to promote this should be explored^(6)^.

College students constitute a significant proportion of the young adult population, making up one-third of people aged 19–24 in Norway^(10)^. Many students are in a dietary transition phase moving away from home and establishing their own family. There is limited knowledge about college students’ diet and dietary behaviours in Norway. This knowledge is needed to substantiate the potential need for interventions targeting diet and lifestyle in preconception years.

The objective of this study was to assess students’ diet in relation to official dietary guidelines provided by the Norwegian Nutrition Council^(1)^ and the Nordic Nutrition Recommendations^(11)^. Further, we aimed to provide assessments of the probability of inadequate micronutrient intake among men and women according to recommended intake (RI), AR and LI, hopefully, helping to identify a potential need and creating a foundation, for interventions to improve diet in this population group during the preconception period.

## Study design

This cross-sectional study, called *StudentKost*, involved a sample of students from the University of Agder, Norway, aged between 18 and 40 years. The average age for men at first birth was 31.8 years in Norway in 2018 and has been above 30 since 1999, so to take this into account we increased the inclusion criteria to 40 years^(12)^. Our recruitment period lasted from the end of October to the beginning of December in 2018. We aimed to recruit 1000 students. In order to aid recruitment, we produced and presented a short promotional video at the university canteens, as well as distributing it in relevant social media. We also distributed flyers in university classrooms and group study rooms. Due to the limited response rate from these recruitment methods, we obtained permission from the university leadership to send out invitations to the university students’ institutional email. The emailed invitation described the study and included a link to the study's web-page, where potential participants could agree to participate and complete the web-based survey. The survey included a validated food frequency questionnaire^(13)^, which was used to assess habitual dietary intake. SurveyXact, a web-based tool for conducting questionnaire-based programs, was used to conduct the survey^(14)^.

### Method

The questionnaire consisted of 209 questions divided into five categories: general information about the participant (5 items), food frequency questions (152 items), eating habits (12 items), motivation and barriers (26 items), physical activity and sedentary behaviour (8 items), and tobacco use (6 items). The general information comprised questions about gender, age, height, weight and education. The food frequency part of the questionnaire divided, e.g. beverages, bread, main courses, fruit and vegetables, into different categories. The response alternatives given in the survey ranged from never eating the specific food item, to eating it every day. There were in total five to seven different frequency categories depending on the nature of the question. The participants had to answer every question in the questionnaire to continue, except questions on height, weight and their email address. The test–retest reproducibility and validity of the Food frequency questionnaire (FFQ) have been assessed and found to have a fair relative validity^(13)^.

Two different programs were used to calculate nutrient intake from the questionnaire, i.e. Python^(15)^ and FoodCalc^(16)^. Python calculates the quantity of each registered food item for every participant in the questionnaire. FoodCalc estimates the nutrient intake by pairing the registered quantity of each food item with its nutrient content from the relevant food item code given in the Norwegian Food Composition Table. This Table provides information concerning the content of nutrients and energy in the most commonly consumed foods in Norway^(17)^. Food intakes are given in grams per day while results for nutrient intakes are presented in grams, milligrams, micrograms or as a percentage of total energy intake depending on the variable of interest. The nutritional calculation does not include supplement use.

Absolute food intakes were compared to official dietary guidelines and the Nordic Nutrition Recommendation (NNR) for the adequacy of nutrient intakes^(18)^. The NNR is based on a comprehensive collaboration of more than 100 experts who reviewed countless research findings in order to determine specific health outcomes of different foods and dietary patterns^(11)^. These assessments have underpinned the national dietary advice, which is used as a reference point in this survey.

### Adequacy of micronutrient intake

Adequacy of micronutrient intake was assessed according to three measures provided by the NNR: the proportion of participants exceeding RI, the proportion of participants with intake below the average requirement (AR) and the proportion of participants with calculated intake below lower intake (LI). RI is estimated to cover individual requirements for 97–98 % of the age- and gender-specific population; hence, intakes above this value imply a low probability of inadequate intake. AR is estimated to cover the requirement for half of the age- and gender-specific population. Intake below this value for a specific micronutrient implies a relatively high probability of inadequate intake^(11)^. LI represents a lower limit of micronutrient intake below which the probability of inadequate intake is very high. Of note, the intake of a specific micronutrient between AR and RI does not guarantee meeting individual needs.

### Statistical analysis

We used IBM SPSS 24.0 to analyse all the data^(19)^. All analyses were carried out stratified by gender, inspecting each variable for deviance from a normal distribution. For comparisons of diet across gender, we used independent sample T-tests, or Mann–Whitney *U* tests depending on the distribution. The significance level was set to *P* < 0⋅05.

## Results

Whereas the invitation was sent to all university students (approximately 13 000), our goal was 1000 participants. A total of 743 participants responded and answered at least one or more questions, equalling to 74 % of our initial goal. Out of these, 617 completed the entire survey. Upon a closer examination of the response patterns among non-completers, we included five who answered each question in the food frequency section of the questionnaire so that their data could be calculated for food and nutrient intake. The final sample included in our analysis was 622. No participants were excluded from the analysis, as no one had an implausible energy intake.

The proportions between women and men in the survey were 71 and 29 %, respectively, with a mean age of 23⋅4 years for women, and 23⋅6 years for men. Table 1 shows height, weight, body mass index (BMI) and educational attainment for the participants. The average BMI was 23⋅8 kg/m^2^ for women and 24⋅4 kg/m^2^ for men. A total of 29 % female and 33 % male respondents were classified with overweight or obesity. In total, 51 % women and 52 % men in this survey had studied for at least 1 year at a university, meaning that a large proportion of the sample were first-time students.

**Table 1.** Age, height, weight, BMI and educational attainment among participating women and men in the StudentKost study

	Women (*n* 444^a^)				Men (*n* 178)			
	Mean	sd	Median	25,75	Mean	sd	Median	25,75
Age, years	23·4	4·5	22·0	20·0, 24·0	23·6	4·0	23·0	21·0, 25·0
Height, cm	168·3	6·4	168·0	164·0, 173·0	182·4	7·4	183·0	178·0, 187·0
Weight, kg	67·4	12·7	65·0	59·0, 74·0	81·2	14·1	80·0	72·0, 89·0
BMI, kg/m^2^	23·8	4·2	23·3	21·2, 25·6	24·4	3·6	23·9	22·2, 25·9
	**Educational attainment**							
	**Women**				**Men**			
	***n***		%		***n***		**%**	
Primary school	3		0·7		0		–	
Secondary school	197		44·4		70		39·3	
Vocational secondary school	15		3·4		14		7·9	
University, less than 4 years	154		34·7		74		41·6	
University, more than 4 years	71		16·0		19		10·7	
Other education	4		0·9		1		0·6	

a Weight is missing 9 (*n* 435) values, and height is missing 2 (*n* 442) values.

### Food consumption

The questionnaire covered a substantial number of food items that were grouped into broader categories for analysis. Table 2 presents the food categories, including fruit, vegetables, fish, whole grain, meat and dairy products. Median reported intake of fruit and vegetables was 209 g/d for women and 148 g/d for men, which is less than half of the recommended 500 g/d. With juice included, the numbers improved to 244 g/d for women and 186 g/d for men. Women had significantly higher fruit and vegetable intake than men in the both cases. For vegetables, 8 % of women met the recommended intake and 5 % of men, while 16 % of women and 8 % of men met the recommendations for fruit intake. For fruits and vegetables combined, a total of 5 % women and 2 % of men met both recommendations; by including juice, this increased to 14 and 9 % for women and men, respectively.

**Table 2.** Intake of different foods and their respective recommendations, in median g/d, if not otherwise stated

Foods, g/d					
	Women		Men		Recommendation^a^
	Median	25, 75	Median	25, 75	
Fish, g/week	225·0	75·0, 397·5	237·5	75·0, 375·0	300–450 g/week
Oily fish, g/week	115·0	40·0, 190·0	115·0	40·0, 190·0	200 g/week
White fish, g/week	100·0	0·0, 175·0	87·5	0·0, 175·0	–
Whole grain^b^	66·6	38·6, 110·1	59·1	37·8, 96·6	70–90 g/d
Red meat^c^	52·7	27·0, 80·6	78·4***	51·1, 115·4	Limit consumption to <500 g/week (approx. 70 g/d)
Fruit	99·1***	50·8, 174·9	59·9	28·9, 126·2	250 g/d
Vegetables	104·9***	59·5, 162·3	74·3	35·9, 116·8	250 g/d
Fruit and vegetables	208·9***	133·5, 331·5	148·0	82·5, 233·3	500 g/d
Juice, orange- or apple-, ml	14·3	0·0, 57·1	14·3	0·0, 57·1	200 ml
Fruit and vegetables, including juice	243·9***	160·9, 387·7	185·7	107·0, 288·8	500 g/d
Total milk, ml	71·4	26·8, 199·6	77·7*	28·6, 227·2	–
Energy-dense dairy products	18·8	9·3, 33·3	22·8*	9·1, 50·0	Reduce intake
Egg	7·0	0·0, 17·6	17·6	0·0, 35·2	–

The StudentKost study.

a Dietary recommendations from the Norwegian Nutrition Council^(1)^.

b Calculated using data from the Norwegian Nutrition Council^(1)^, product web sites^(20)^, and the Norwegian Food Composition Table^(21)^.

c Calculated using information from manufacturers websites^(22–26).^

Significance level for differences between genders: *P < 0·05, ***P < 0·001.

Mean fish intake was 225 g/week for women and 238 g/week for men, with oily fish contributing 115 g/week for both genders. This is substantially lower than the recommended 300–450 g/week of fish in general, and 200 g/week of oily fish. Approximately 17 % did not report eating any fish. Additionally, both genders had a lower-than-recommended intake of 70–90 g/d of whole-grain products; women consumed 67 g/d, while men consumed 59 g/d. The consumption of red meat added up to 53 g/d for women and 78 g/d for men. Men ate significantly more red meat than women. It is recommended to limit the consumption of red and processed meat to less than 500 g/week or approximately 70 g/d. Around 17 % of women in this sample did not consume red meat, while less than 2 % of men did the same. A total of 2·3 % women and 1·7 % of men did not eat any kind of meat. Total milk intake added up to 71 ml/d for women and 78 ml/d for men.

### Energy intake

Table 3 presents energy intake and macronutrient contribution. Mean energy intake was 8·6 MJ/d for women and 9·5 MJ/d for men. On average, carbohydrates contributed to 44·5 E% for women, of which 5·8 E% came from added sugar. For men, energy from carbohydrates amounted to 42·1 E%, of which 5·9 E% came from added sugar. Thus, both genders had sugar intake well within the recommended limit of 10 E%. The main contributor to sugar intake was soda, with a median intake of 35·7 ml/d for both genders. Dietary fibre intake was lower than the recommended 25–35 g/d for both genders but significantly higher for women than for men (22·1 v. 20·3 g/d).

**Table 3.** Energy intake and macronutrient contribution to energy intake compared to the Nordic Nutrition Recommendations by gender, presented as E% if not otherwise stated.

	Women				Men				NNR (E%)
	Mean	sd	Median	25,75	Mean	sd	Median	25,75	
Energy, kcal	2045	719	1993	1535, 2376	2274	739***	2203	1736, 2780	–
Energy, MJ/d	8·6	3·0	8·3	6·4, 10·0	9·5	3·1*	9·2	7·3, 11·7	–
Carbohydrates	44·5	6·7***	44·2	40·5, 48·3	42·1	7·1	41·9	38·3, 45·9	45–60
Added sugar	7·1	5·2	5·8	3·9, 8·4	7·0	5·5	5·9	3·4, 8·7	<10
Dietary fibre, g/d	23·7	10·6	22·1	15·7, 29·2*	21·9	11·2	20·3	14·7, 26·8	25–35 g/d
Total fat	33·4	5·6	33·5	30, 36·9	34·3	5·8	34·3	30·7, 38·1	25–40
SFA	12·1	3·0	12·1	10·3, 14·0	12·7	3·2*	12·5	10·5, 14·2	<10
TFA	0·3	0·1	0·3	0·2, 0·4	0·3	0·2	0·3	0·2, 0·4	As low as possible
MUFA^|^	12·9	2·4	12·7	11·3, 14·4	13·3	2·5	13·1	11·6, 15·1	10–20
PUFA	5·0	1·1	4·9	4·3, 5·7	5·0	1·3	4·8	4·1, 5·7	5–10
Omega 3	1·1	0·4	1·0	0·8, 1·3	1·1	0·5	1·0	0·7, 1·4	At least 3 E%
Omega 6	3·8	0·8	3·8	3·3, 4·4	3·8	0·9	3·7	3·2, 4·4	
Protein	17·4	3·1	17·3	15·3, 19·3	17·9	3·9	17·6	15·8, 19·6	10–20
Alcohol	2·4	2·5	1·5	0·6, 3·5	3·8	3·8	3·4	0·7, 6·0***	<5

The StudentKost study.

NNR, Nordic Nutrition Recommendations; SFA, saturated fatty acids; TFA, trans fatty acids; ^|^MUFA, monounsaturated fatty acids; PUFA, polyunsaturated fatty acids.

Significance level for differences between genders: *P < 0·05, ***P < 0·001.

Fat contributed on average 33·4 E% for women and 34·3 E% for men. Both genders met the recommended intake of omega 3 and 6 of at least 3 % of the total energy intake combined. Saturated fatty acids contributed to 12·1 E% for women and 12·7 E% for men which is higher than the recommended upper limit of 10 E% for both genders. Monounsaturated fats contributed to 12·9 E% for women and 13·3 E% for men. Polyunsaturated fats made up 5·0 E% for both genders, which is at the lower end of the recommended 5–10 E%. Protein contributed to a total of 17·4 E% for women and 17·9 E% for men, which is within the recommended range of 10–20 E%. Finally, alcohol amounted to an average of 1·5 E% for women and 3·4 E% for men. In this sample, 16·7 % reported not drinking any alcohol.

### Micronutrients

Tables 4 and 5 present mean and median micronutrient intake compared to the recommended intake (RI), the average requirement (AR) and lower intake level (LI) for both genders, respectively. On average, men had a significantly higher intake of most micronutrients compared to that of women. However, as the recommended intake is higher for men regarding most micronutrients, intake was still suboptimal for some nutrients. Men had a significantly higher intake of vitamin B_6_, vitamin B_12_ and vitamin E, iodine and calcium than women. Women, on the other hand, had a significantly higher intake of vitamin C than men. Importantly, the listed intakes do not include the use of supplements. Participants’ use of supplements is described below.

**Table 4.** Micronutrient intake per day compared to NNR and related to recommendations in percentage.

Women										
Micronutrients	Mean	sd	Median	25th–75th percentile	NNR2012 recommendation			Percentage of participants^a^		
					RI^b^	AR^b^	LI^b^	≥RI	<AR	<LI
Vitamin A, μg	930	763	715	463, 1089	700 μg	500 μg	400 μg	52 %	29·5 %	16 %
Vitamin D, μg	5·5	3·6	4·7	3·0, 7·4	10 μg	7·5 μg	2·5 μg	12 %	76·1 %	18 %
Vitamin E, mg	12·0	5·3	10·9	8·4, 14·3	8 mg	5 mg	3 mg	79 %	2·7 %	0·5 %
Thiamine, mg	1·5	0·6	1·4	1·0, 1·8	1·1 mg	0·9 mg	0·5 mg	71 %	14·2 %	0·5 %
Riboflavin, mg	1·9	1·1	1·7	1·2, 2·2	1·3 mg	1·1 mg	0·8 mg	71 %	18·7 %	4·3 %
Niacin, mg	23·0	13·4	20·3	14·9, 27·0	15 mg	12 mg	9 mg	75 %	11·9 %	2·7 %
Vitamin B_6_, mg	2·0	0·8	1·8	1·4, 2·3	1·2 mg	1·0 mg	0·8 mg	86 %	6·8 %	1·8 %
Folate, μg	281	121	257	186, 360	400 μg	200 μg	100 μg	15 %	30·6 %	1·4 %
Vitamin B_12_, μg	6·2	3·5	5·5	3·7, 8·0	2 μg	1·4 μg	1·0 μg	94 %	2·0 %	1·6 %
Vitamin C, mg	106·0	68·6	87·9	58·9, 132·0	75 mg	50 mg	10 mg	60 %	16·9 %	0
Calcium, mg	802	404	720	532, 991	800 mg	500 mg	400 mg	40 %	21·4 %	11·9 %
Phosphorus, mg	1654	638	1558	1182, 1969	600 mg	450 mg	300 mg	100 %	0	0
Magnesium, mg	354	134	335	256, 423	280 mg	–	–	68 %	–	–
Potassium, g	3·6	1·3	3·5	2·7, 4·4	3·1 g	–	1·6 g	0	–	–
Sodium, g	3·5	1·6	3·2	2·3, 4·2	2·4 g	–	–	0	–	–
Iron, mg	10·5	4·0	10·2	7·5, 12·7	15 mg	10 mg	10 mg^a^	12 %	46·6 %	46·6 %
Zinc, mg	11·1	4·1	10·6	8·2, 13·4	7 mg	5 mg	4 mg	86 %	2·9 %	0·9 %
Copper, mg	1·2	0·5	1·2	0·8, 1·5	0·9 mg	0·7 mg	0·4 mg	97·5 %	12·2 %	2·5 %
Selenium, μg	47·0	22·8	43·3	30·2, 60·3	50 μg	30 μg	20 μg	38 %	24·1 %	8·3 %
Iodine, μg	134	83	114	73, 165	150 μg	100 μg	70 μg	32 %	42·3 %	21·6 %

The StudentKost study.

NNR: Nordic Nutrition Recommendations; RI: recommended intake; AR: average requirement; LI: lower intake level.

a Out of total sample.

b Age group 18–30 was used as the choice of reference intake.

**Table 5.** Micronutrient intake per day compared to NNR and related to recommendations in percentage

Men										
Micronutrients	Mean	sd	Median	25^th^–75^th^ percentile	NNR2012 recommendation			Percentage of participants^a^		
					RI^b^	AR^b^	LI^b^	≥RI	<AR	<LI
Vitamin A, μg	963	779	762	463, 1137	900 μg	600 μg	500 μg	39 %	36·5 %	28·1 %
Vitamin D, μg	6·0	3·7	5·5	3·3, 8·0	10 μg	7·5 μg	2·5 μg	15 %	68·5 %	14 %
Vitamin E, mg	13·3	6·0	12·3	9·0, 16·8	10 mg	6 mg	4 mg	70 %	7·9 %	1·7 %
Thiamine, mg	1·6	0·6	1·6	1·1, 2·0	1·4 mg	1·2 mg	0·6 mg	58 %	31·5 %	1·7 %
Riboflavin, mg	2·2	1·1	2·0	1·5, 2·6	1·6 mg	1·4 mg	0·8 mg	73 %	19·1 %	4·5 %
Niacin, mg	26·3	13·1	24·6	17·8, 31·4	19 mg	15 mg	12 mg	73 %	14·0 %	6·7 %
Vitamin B_6_, mg	2·2	0·8	2·0	1·6, 2·6	1·5 mg	1·3 mg	1·0 mg	80 %	13·5 %	5·1 %
Folate, μg	275	119	265	188, 346	300 μg	200 μg	100 μg	37 %	28·7 %	2·8 %
Vitamin B_12_, μg	7·2	3·7	6·5	4·5, 9·1	2 μg	1·4 μg	1·0 μg	96 %	1·7 %	1·1 %
Vitamin C, mg	88·5	57·5	73·4	48·6, 113·5	75 mg	60 mg	10 mg	48 %	37·6 %	0·6 %
Calcium, mg	887	443	790	568, 1088	800 mg	500 mg	400 mg	49 %	20·2 %	11·2 %
Phosphorus, mg	1805	665	1779	1265, 2245	600 mg	450 mg	300 mg	99 %	0	0
Magnesium, mg	362	140	344	255, 438	350 mg	–	–	49 %	–	–
Potassium, g	3·7	1·4	3·6	2·6, 4·3	3·5 g	–	1·6 g	0	–	–
Sodium, g	4·1	1·8	4·0	2·8, 5·0	2·4 g	–	–	0	–	–
Iron, mg	11·0	4·3	10·9	8·0, 14·0	9 mg	7 mg	7 mg	62 %	18·5 %	18·5 %
Zinc, mg	12·7	4·5	12·8	9·2, 15·6	9 mg	6 mg	5 mg	77 %	5·1 %	1·7 %
Copper, mg	1·2	0·5	1·1	0·8, 1·5	0·9 mg	0·7 mg	0·4 mg	70 %	13·5 %	2·3 %
Selenium, μg	54·6	24·2	52·7	38·6, 67·7	60 μg	35 μg	20 μg	37 %	18·0 %	6·2 %
Iodine, μg	143	78	133	91, 184	150 μg	100 μg	70 μg	41 %	33·1 %	16·9 %

The StudentKost study.

NNR: Nordic Nutrition Recommendations; RI: recommended intake; AR: average requirement; LI: lower intake level.

a Out of total sample.

b Age group of 18–30 years was used as the choice of reference intake.

In this study, 88 % of women and 85 % of men did not reach the RI of vitamin D. The RI of vitamin A was not reached by 48 % women and 61 % of men. Approximately 85 % of female students did not reach the RI of folate, 88 % for iron and 68 % for iodine. Regarding male students, a total of 63 % did not reach the RI of folate, 38 % for iron and 59 % for iodine. The Norwegian Directorate of Health recommends reducing the salt intake to a maximum of 5 g/d^(1)^, while the NNR recommends a maximum of 6 g/d^(11)^. In this sample, both genders consumed more salt than recommended, with a mean intake of 8·7 g/d among women and 10·2 g/d among men.

#### Probability of inadequate micronutrient intake

We also assessed the proportions of participants with *minimal probability* of inadequate intake (total intake of the respective micronutrient at or above the recommended intake), *relatively high probability* of inadequate intake (the total intake of the respective micronutrient lower than the average intake) and *very high probability* of inadequate intake (the total intake of the respective nutrient below the lower intake), respectively (Tables 4 and 5). The probability of inadequate intake was low for vitamin E, B-vitamins other than folate, phosphorus, zinc, copper, riboflavin, and niacin (proportion of students with intake at or above RI ≥70 %) (Tables 4 and 5). The proportion of female participants with intake less than the average requirement, and thus, relatively high probability of inadequate micronutrient intakes was substantial for vitamin A (30 %), vitamin D (76 %), folate (31 %), calcium (21 %), iron (47 %), selenium (24 %) and iodine (42 %) (Table 4). A smaller, but still substantial, proportion of female students had a *very high probability* of inadequate intake of vitamin A (16 %), vitamin D (18 %), iron (47 %) and iodine (22 %). The proportion of male participants with a relatively high probability of inadequate micronutrient intakes was substantial for vitamin A (37 %), vitamin C (38 %), vitamin D (69 %), folate (29 %), calcium (20 %) and iodine (33 %) (Table 5). A smaller but still substantial proportion of male students had a *very high probability* of inadequate intake of vitamin A (28 %), vitamin D (14 %), iron (19 %) and iodine (17 %).

### Supplements

A total of 17·6 % female and 16·3 % male students reported taking omega-3 or cod-liver oil capsules every day. In this sample, 16·9 % of women and 12·4 % of men reported taking multivitamins, or multivitamins with minerals, every day. Although folate intake was lower than recommended for women, only 2·5 % of the sample reported taking folate supplements every day. Furthermore, 95 % of all women reported never taking folate supplements. Men showed similar numbers with 96 % never taking folate supplements, and 2 % taking it every day. Similarly, only 6 % of women took iron supplements every day, while 4 % of men did the same. Among vitamin A, C and D supplements, vitamin D supplements were consumed most frequently with 12 % of both women and men taking it every day.

## Discussion

Our main findings are that college students, both male and female, have a somewhat suboptimal diet compared to Norwegian dietary guidelines. Fruits, vegetables, fish, milk and whole-grain products are all significant contributors to a varied and healthy diet, especially during the preconception period^(27)^. Fruit and vegetable intake was substantially lower than recommended and a lot lower than reported in Norkost 3^(2)^. The consumption of both whole grain and fish were low for both genders, with mean intake just above half of the recommended intake of fish. This may partly explain the low vitamin D and iodine intake.

WHO estimates that approximately 1·7 million or 2·8 % of deaths worldwide are related to low consumption of fruit and vegetables^(28)^, and low fruit consumption was the most significant individual dietary risk factor in 2010^(29)^. A high intake of fruit and vegetables has been associated with a reduced risk of Cardiovascular disease (CVD), cancer and other major health problems^(30–32)^. Eating fish 2–4 times a week may reduce the risk of heart conditions such as Coronary heart disease (CHD) and heart failure^(33,34)^. According to the Global Burden of Disease study from 2019, a high intake of sodium, and a low intake of whole grains and fruits were the leading dietary risk factors for death and disability-adjusted life years, of which low intake of whole grain was the leading risk among young adults^(35)^. Dietary fibre and whole-grain products connect to a multitude of health benefits, among them being a lower risk of high blood pressure and a lower concentration of Low-density lipoprotein-cholesterol (LDL), reduced risk of all-cause mortality as well as cardiovascular disease and cancer mortality^(36–38)^, of which high blood pressure before pregnancy is considered a risk factor for pre-eclampsia^(39)^. Milk and dairy products contribute between 60 and 80 % of the iodine intake in the Norwegian diet and are considered its most important source^(40)^.

Only 12 % complied with the recommendation of ‘5-A-Day’, or 500 g/d, even when juice was included. In Norkost 3, from the entire sample, a total of 25 % of women and 22 % of men complied with the recommendation on fruit and vegetables^(2)^, while results from the British National Diet and Nutrition Survey (NDNS) showed that 32 % women and 29 % men aged 19-64 satisfied the ‘5-A-Day’ recommendation^(41)^. Results from the Dutch National Food Consumption Survey (DNFCS), however, showed that among 703 participants aged 19–30 years, less than 5 % satisfied the recommended 200 g vegetables per day^(42)^. Consuming 500 g or more fruit and vegetables per day has been associated with reduced risk of cardiovascular diseases^(43)^, such as coronary heart disease^(44)^, and Jian *et al*. found the lowest risk for cardiovascular disease among those consuming 800 g or more fruit and vegetables per day^(43)^.

Macronutrient contribution to energy intake seems to be in line with the recommendations from the NNR within a few percentages^(11)^. While sugar intake was well within recommended limits for most participants, one unfavourable finding was the low intake of dietary fibre for both genders. Countless studies have investigated the importance of dietary fibre, of which many have found inverse associations with risk of CVD and all cancers^(45–47)^. Dietary fibre also promotes a healthy microbiota^(48,49)^. These findings encourage further investigation into young adults’ diet as an important determinant for long-term health.

In this limited sample of college students, we found high proportions of participants with a relatively high probability of inadequate intake of vitamin A, vitamin D, vitamin C (male), folate, calcium, iron, selenium and iodine. The suboptimal intake of these nutrients is a direct consequence of the low intake of core foods such as fruits, vegetables, whole grain, fish and dairy products in the present study. However, a low intake does not translate directly to compromised nutritional status of the particular micronutrients, this being especially relevant to vitamin D status since vitamin D can be synthesized in the skin by the ultraviolet radiation of its precursor 7-dehydrocholesterol^(50,51)^. Intake of folate, iron and iodine was suboptimal among female students. Folate (vitamin B_9_) is considered a critical nutrient during the periconception period^(52,53)^, as a low intake has been associated with increased risk of Spina Bifida and other neural tube defects for the foetus^(54)^. Sufficient iron intake during preconception and reproductive years are vital in building capacity for the large requirements during pregnancy and the inherent risk of iron deficiency and anaemia^(55)^. Lastly, a low intake of iodine increases the risk of goitre, and a sufficient intake is necessary to ensure proper foetal development from early pregnancy onwards^(56)^. Iodine also has essential roles in several bodily regulatory functions, like metabolic rates, the immune system and the central nervous system^(57)^.

We found gender differences in diet among students, with female students in general eating healthier than male students. This raises concern about male awareness of the importance of diet, as new research also highlights the importance of a healthy paternal diet during the preconception period^(4–6)^. We expected some gender differences due to differences in energy intake, but the higher absolute intake of fruit and vegetables, whole grain, dietary fibre and proportion of energy from carbohydrates among women were surprising. Results from Norkost 3 and the DNFCS showed no significant difference in the intake of vegetables between genders, while this study showed a difference in favour of women^(2)^.

### Study strengths and limitations

This study aimed to assess the dietary intake and nutritional adequacy of young adults through a dietary questionnaire, here represented by students in their preconception years. The questionnaire was designed so that participants had to answer all questions, except height, weight and their email address. The advantage of using this method was that we got very few missing values in our data. On the other hand, we do not know what questions caused the remaining 121 participants not to complete the survey. Using an FFQ has its pros and cons. The FFQ was very comprehensive, and we were able to collect a large amount of information in a short period, but as the food items are set beforehand, we missed some diet variables which could have had an impact on our results. Most noticeably, specific vegetables and berries, which were mentioned by a good number of participants in the comment section on the end of each part of the questionnaire. Misreporting in dietary surveys is common^(58)^. If overreporting of healthy foods or underreporting of unhealthy foods are prevalent in the present study, challenges related to diet may be even more substantial than our findings suggest, and public health initiatives aimed at diets in this population of young adults would be even more relevant. Red meat intake may be underestimated because we did not include taco in the calculation, which is a popular dish that is eaten by 13% of the population at least once a week^(59)^. As a consequence, we cannot make definitive conclusions on meat intake, which might have an impact on iron intake.

There might also be some limitations in the software that was used. It is difficult to convert the number of portions to a total of grams eaten, as the calculations are based on a report on standard portion sizes from the Norwegian Food Safety Authority, the University of Oslo, and the Norwegian Directorate of Health^(60)^. How you define one portion is based on your personal preference, so this leaves room for error. The portions used are the average portions sized for adults in Norway^(60)^.

We contacted all enrolled university students by way of their institutional email in the recruitment to this study; hence, the low response rate compromises the generalisability of our findings. Although all students received an email, we do not know if they check their institutional email consistently. We do not know to which degree the diet of our study population differs from the rest of the accessible population at UiA or students at other universities in Norway. Moreover, the sample might not be an accurate representation of the entire young adult population which must be considered when interpreting this data. Still, the best data for this age group in Norway are currently from the national dietary surveys, including 18–70 years, with only 281 participants being 18–29 years old, as well as being from 2011^(2)^.

Since we targeted students, educational attainment in this sample is at a higher percentage than the average of the Norwegian population, where 33·4 % aged 16 years or older have attained higher education^(61)^. However, it is similar to the education level of the national study Norkost 3^(2)^. As there are a lot more women than men in the sample, the data on women may be more representative than the data on men. If anything, due to education attainment and self-selection for the study, our sample is likely to be more health-conscious than the broader population, making our findings regarding dietary weaknesses of concern in a public health perspective.

## Conclusion

This study indicates dietary weaknesses among university students and potential inadequacy for several micronutrients. Gender differences indicated a healthier diet in favour of female students, including higher consumption of fruits, vegetables, and fibre, and lower red meat and high energy-dense dairy products consumption. A substantial proportion of both male and female students had a relatively high probability of inadequate intake of several micronutrients, including vitamin A, vitamin D, folate, iron and iodine.

Our findings have limitations and should be interpreted with caution. However, the fact that our sample might be a selected group with an above-average interest in diet and nutrition highlights the need for attention and public health nutrition actions towards this group. Our results call for more representative dietary assessment studies to be performed among young adults to inform further action towards a healthier diet during the preconception period.
